# Prion protein is essential for the RE1 silencing transcription factor (REST)-dependent developmental switch in synaptic NMDA receptors

**DOI:** 10.1038/s41419-018-0576-z

**Published:** 2018-05-10

**Authors:** Zhiqi Song, Wei Yang, Guangyu Cheng, Xiangmei Zhou, Lifeng Yang, Deming Zhao

**Affiliations:** 1 The State Key Laboratories for Agrobiotechnology, Key Lab of Animal Epidemiology and Zoonosis, Ministry of Agriculture, National Animal Transmissible Spongiform Encephalopathy Laboratory, College of Veterinary Medicine, China Agricultural University, 100193 Beijing, China; 2 Institute of Laboratory Animal Sciences, Chinese Academy of Medical Sciences (CAMS) and Comparative Medicine Center, Peking Union Medical Collage (PUMC), Key Laboratory of Human Disease Comparative Medicine, Ministry of Health, 100021 Beijing, China; 3 Hebei Institute of Animal Science and Veterinary Medicine, 071000 Baoding, China

## Abstract

It is important that the correct amounts of GluN2 subunits are maintained, as they determine NMDAR functional properties, which are crucial to neuronal communication, synaptogenesis and cognitive function. The transcriptional repressor RE1 silencing transcription factor (REST) is critical for the postnatal developmental switch in NMDARs. However, the mechanisms triggering REST and the link between NMDARs and REST are unclear. Here we show a new physiological essential role for cellular prion protein (PrP^C^) in REST-dependent homeostasis and the developmental switch of NMDARs. REST and REST-associated proteins were overactivated in the hippocampi of *Prnp* knockout mice (*Prnp*^*0/0*^) compared with wild-type *Prnp* (*Prnp*^*+/+*^) mice. This coincided with the disruption of the normal developmental switch from GluN2B-to-GluN2A in vivo. PrP^C^ co-located with REST under physiological environments and mediated the translocation of REST in conditioners of NMDARs in vitro in *Prnp*^*+/+*^ hippocampal neurons. Regardless of whether REST was knocked down or overexpressed, deletion of PrP^C^ not only disrupted REST-mediated distribution of mitochondria, but also prevented REST-regulated expression of GluN2B and GluN2A in *Prnp*^*0/0*^. Importantly, these effects were rescued after overexpression of full-length PrP^C^ through restoration of NMDAR2 subunits and their distributions in dendritic processes in *Prnp*^*0/0*^. Consistently, knockdown of PrP^C^ in *Prnp*^*+/+*^ had a similar effect on *Prnp*^*0/0*^. Furthermore, PrP^C^ colocalized with both GluN2B and GluN2A in *Prnp*^*+/+*^. For the first time, we demonstrate that PrP^C^ is essential for REST-regulated NMDARs. Confirming the regulation of NMDAR-modulating mechanisms could provide novel therapeutic targets against dysfunctions of glutamatergic transmission in the nervous system.

## Introduction

*N*-methyl-d-aspartate receptors (NMDARs) are glutamate-gated ion channels critical for synaptogenesis and neuronal communication^1^. These heterotetrameric channels are formed by the assembly of two obligatory GluN1 and two GluN2/GluN3 subunits^2,3^. NMDARs subunit composition is plastic and diverse, leading to abundant receptor subtypes, each with its own biophysical and pharmacological properties^1^. The subunit isoform of NR2 (GluN2) is a key determinant of the functional capabilities of NMDARs, including activation, deactivation and desensitization kinetics^4^. A developmental switch from containing primarily GluN2B-to-GluN2A occurs during postnatal development in NMDARs^5^. This switch, as well as the correct GluN2A/CluN2B ratio, is critical for neural circuitry^6^, hippocampus-dependent learning^7^, plasticity-induced alpha-amino-3-hydroxy-5-methyl-4-isoxazolepropionic acid (AMPA) receptors^8^ and spine growth^9^. A previous report has shown that the transcriptional repressor element 1-silencing transcription factor (REST), also known as neuron-restrictive silencer factor^10^, participates in the postnatal switch in synaptic NMDAR subunit by decreasing GluN2B expression through epigenetic remodeling of *Grin2b* at rat hippocampal synapses. During postnatal development, REST shows both target and temporal characteristics in differentiated neurons for GluN2B^11^. REST acts as a multiple hub in coordination with other factors regulating the multiple aspects of neurogenesis and preserving the distinct neural phenotype^10^. However, as a switch from primarily GluN2B-to-GluN2A, the mechanisms that trigger REST expression in differentiated neurons, and the following long-term increase in GluN2A expression during postnatal development, are still unclear.

Although under normal conditions NMDA receptors mediate important physiological functions such as learning and memory, they also take part in glutamate excitotoxicity, which can occur in response to ischemia and is related to many neurodegenerative diseases, including Alzheimer's Disease (AD)^12^. Hence, strict regulation of NMDAR activity plays a key role in homeostasis by preventing excitotoxicity^13^. It is well established that misfolded forms of cellular prion protein (PrP^C^) transform into the β-sheet-rich, aggregate-prone scrapie conformation (PrP^Sc^), resulting in several progressive fatal diseases, known as prion diseases. These transmissible spongiform encephalopathies include bovine spongiform encephalopathy, scrapie, Creutzfeld–Jakob disease, Gerstmann–Straussler–Scheinker syndrome in humans^14,15^. Although research has been conducted exploring the harmful effects of misfolded or aggregated prion proteins, the physiological roles of PrP^C^ remain only partly understood.

Recent studies have demonstrated that PrP^C^ also communicates with NMDARs; PrP^C^-deficient mice exhibit enhanced NMDAR-dependent neuronal excitability and are more susceptible to NMDA-induced excitotoxicity^16^. Research has cumulatively demonstrated that the absence of PrP^C^ increased NMDAR glycine affinity, resulting in persistent NMDAR activity after prolonged agonist treatment^17^. PrP^C^ and GluN2D are found in the same protein complex and colocalize in hippocampal neurons^16^. However, in the absence of PrP^C^, it is still not clear whether other subunits of NMDARs are altered. Therefore, more details of PrP^C^-mediated regulation of NMDARs need to be revealed.

Here, we explore the relationship between PrP^C^ and REST-dependent developmental switch from GluN2B-to-GluN2A. Our data show that a lack of PrP^C^ gives rise to REST overexpression and disorder of REST-associated proteins in the hippocampus of neonatal mice. REST-dependent epigenetic remodeling of the developmental switch of NMDARs is also suppressed in vivo. Comparing wild-type (WT; *Prnp*^*+/+*^) and *Prnp* knockout (*Prnp*^*0/0*^) mice primary cultured hippocampal neurons, we found that PrP^C^ not only affects the translocation of REST, but also partially mediates REST-regulated mitochondria distribution and the developmental switch in synaptic NMDARs. The adverse effects induced by overactivated REST in *Prnp*^*0/0*^ neurons are recapitulated by overexpression of exogenous PrP^C^. Thus, we demonstrate a novel functional role for native PrP^C^ as an essential modulator of REST-dependent NMDARs activity, and provide more evidence to support the hypothesis that PrP^C^ is a regulator of NMDARs.

## Results

### Overexpression of REST and alterations of REST-associated proteins in the hippocampi of postnatal *Prnp*^*0/0*^ mice

**Fig. 1:**
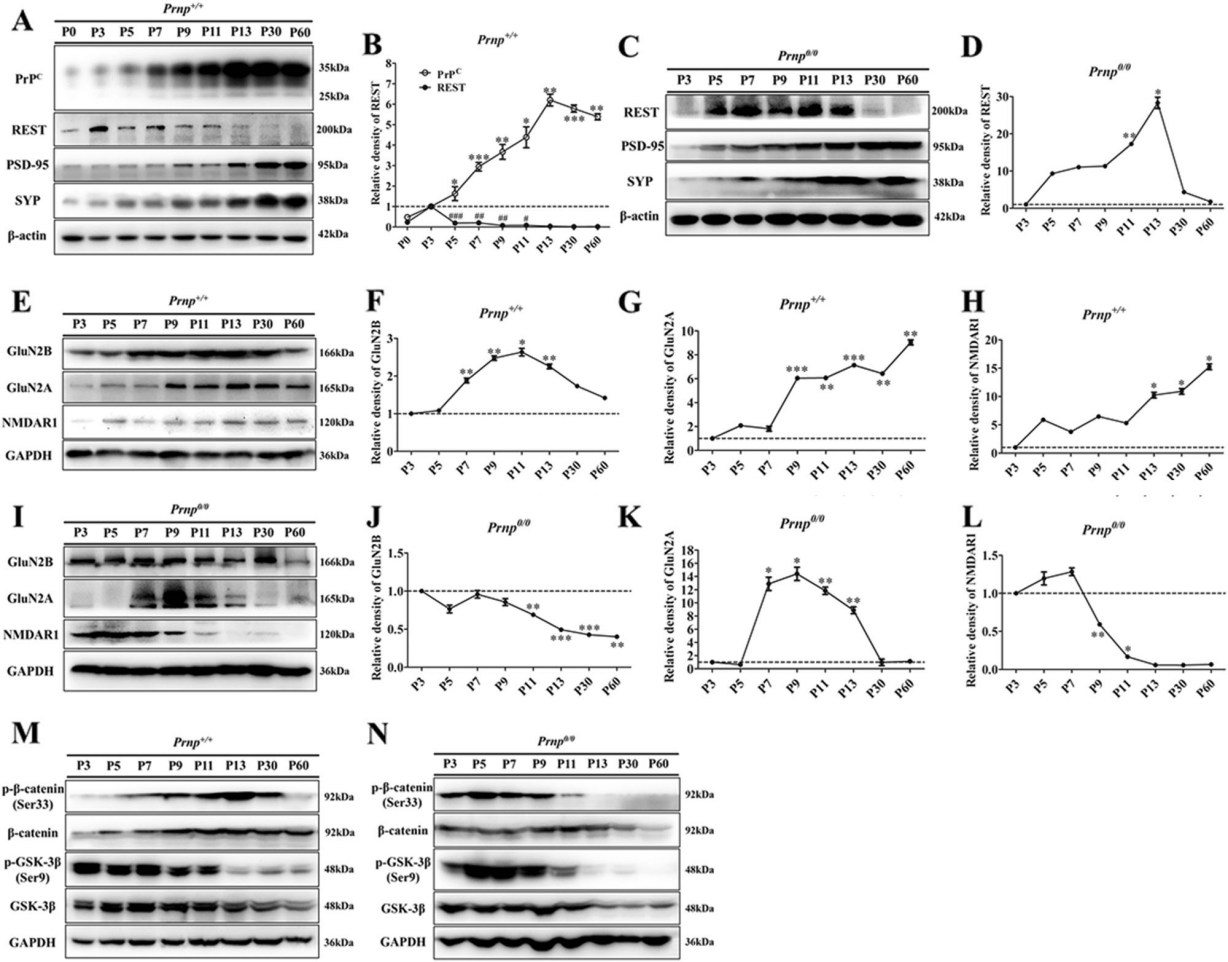
Western blot (WB) analyses of PrP^C^ (in wild-type (WT) only), REST, PSD-95, SYP, GluN2B, GluN2A, NMDAR1, total and phosphorylated β-catenin and GSK3β during WT *Prnp* (*Prnp*^*+/+*^) and PrP-null (*Prnp*^*0/0*^) mice hippocampal postnatal development. **a**, **e**, **m** WB results in whole hippocampal lysates of WT mice (*n* = 6). **c**, **i**, **n** WB results in whole hippocampal lysates of *Prnp*^*0/0*^ mice (*n* = 6). **b** Quantitative analyses of (**a**). **d** Quantitative analyses of (**f**). **f**–**h** Quantitative analyses of (**e**). **j**-**l** Quantitative analyses of **i**. Immunoblot density in **b** and **d** normalized to β-actin. Immunoblot density in **f**–**h** and **j**–**l** normalized to GAPDH. All values were normalized (dashed lines) relative to corresponding data at P3 in each group. Data are presented as means ± SD (*n* = 6). *P < 0.05; **P < 0.01; ***P < 0.001 vs corresponding data at P3

In almost all tissues, including the brain, epigenetic modification of chromatin is an important modulator of gene expression^18,19^. First, we confirmed the developmental expression of PrP^C^ in the hippocampus of *Prnp*^*+/+*^ neonatal mice. Consistent with previous reports^20,21^, PrP^C^ levels increased during the first two postnatal weeks, reached a peak at P13 and were slightly diminished in the adult (Fig. 1a, b). Second, we tested and compared postnatal expression of REST, postsynaptic density 95 kDa protein (PSD-95), synaptophysin (SYP), GluN2B, GluN2A and NMDAR1 in the hippocampal homogenates of *Prnp*^*+/+*^ and *Prnp*^*0/0*^ neonatal mice.

In the WT group, contrary to the tendency of PrP^C^, a transient, but marked increase in REST abundance occurred at postnatal day 3 (P3; Fig. 1a, b), with a subsequent, and constant, abundance of GluN2B protein from P7 until P13. REST abundance then declined ~2.25-fold (relative to P3) to a level that persisted until adulthood (Fig. 1e, f). GluN2A protein was barely detectable at P3, and its levels progressively increased ~6.43-fold by P30 relative to that at P3, and continued to increase until P60 (nearly ninefold) (Fig. 1e, g). NMDAR1 levels gradually increased in a similar way to GluN2A (Fig. 1e, h).

**Fig. 2:**
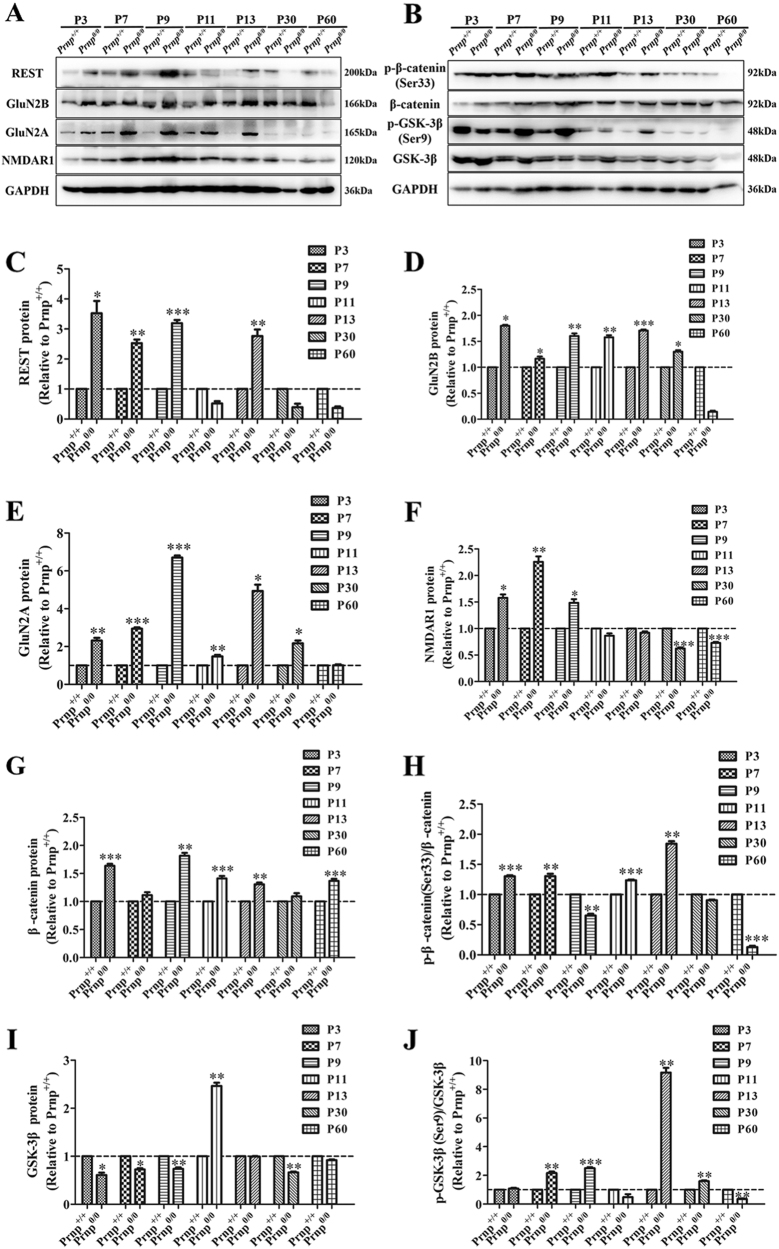
WB analyses of REST and associated proteins to compare *Prnp*^*+/+*^ (*n*  =  6) with *Prnp*^*0/0*^ (*n*  =  6) mice hippocampal postnatal development. **a** Immunoblot of REST, GluN2B, GluN2A, and NMDAR1. **b** Immunoblot of total and phosphorylated β-catenin and GSK3β proteins. **c**–**f** Quantitative analyses of (**a**). **g**–**j** Quantitative analyses of (**b**). Immunoblot density in **c**–**g** and **i** normalized to GAPDH and expressed as the ratio to GAPDH density. Immunoblot density in **h** and **j** showing the quantification of β-catenin (Ser33)/total β-catenin protein, GSK3β (Ser9)/total GSK3β protein, total β-catenin and GSK3β protein normalized to GAPDH. All values were normalized (dashed lines) relative to corresponding data in the *Prnp*^*+/+*^ group. Data are presented as means ± SD (*n* = 6). *P < 0.05; **P < 0.01; ***P < 0.001 vs *Prnp*^*+/+*^

In the *Prnp*^*0/0*^ group, REST expression significantly increased after P5 (~9.32-fold) and the highest levels were seen at P13 (~28.32-fold) relative to P3 (Fig. 1c, d). Although GluN2B levels progressively declined after P3 (Fig. 1i, j), GluN2B showed higher expression from P3 to P30 compared with the WT group (Fig. 2a, d). GluN2A was barely detectable before P5, but its expression markedly increased from P7 to P13 (Fig. 1i, k). NMDAR1 was strongly expressed from P3 to P7 and decreased to undetectable levels at P60 (Fig. 1i, l).

Compared with WT controls, *Prnp*^*0/0*^ mice showed significantly higher expression of REST (Fig. 2c), GluN2B (Fig. 2d) and GluN2A (Fig. 2e) during postnatal development (Fig. 2a). NMDAR1 expression was higher from P3 to P9 but decreased remarkably after P30 (Fig. 2f). Levels of SYP and PSD-95, the pre- and postsynaptic markers, showed a consistent upward trend and were not significantly different between the two groups.

We recently demonstrated that β-catenin were expressed in parallel with REST under physiological^22^ and pathological conditions in LRP6-Wnt-β-catenin signaling pathways^23,24^. Conversely, total GSK3β protein has an inverse relationship with REST^24^. Our results reveal that: (1) total β-catenin and p-β-catenin (Ser552)/total β-catenin protein had different expression patterns between WT and PrP-null mice: the former remained relatively high after P7 (Fig. 1m, Fig. S1E, and Fig. S1F), whereas the latter was more highly expressed at the beginning of P3 (Fig. 1n, Fig. S1I, and Fig. S1J). (2) Comparing WT and *Prnp*^*0/0*^ mice, total β-catenin was still more highly expressed in the *Prnp*^*0/0*^ group, and p-β-catenin (Ser552)/total β-catenin protein was generally more highly expressed in the latter, except at P9, but significantly decreased at P60 (Fig. 2b, g, h). (3) Although total GSK3β protein and p-GSK3β (Ser9)/total GSK3β protein had similar expression patterns in both groups (Fig. 1m, Fig. S1G, and Fig. S1H; Fig. 1n, Fig. S1K, and Fig. S1L), GSK3β protein levels were lower in *Prnp*^*0/0*^ mice except at P11, and p-GSK3β (Ser9)/total GSK3β protein was higher in the latter until P60. These findings are consistent with the state of REST in different groups.

Overall, *Prnp*^*0/0*^ mice exhibited REST overexpression and disordered postnatal developmental switching from GluN2B-to-GluN2A, together with overactivation of β-catenin and suppression of GSK3β.

### PrP^C^ is essential for REST functional translocation

**Fig. 3:**
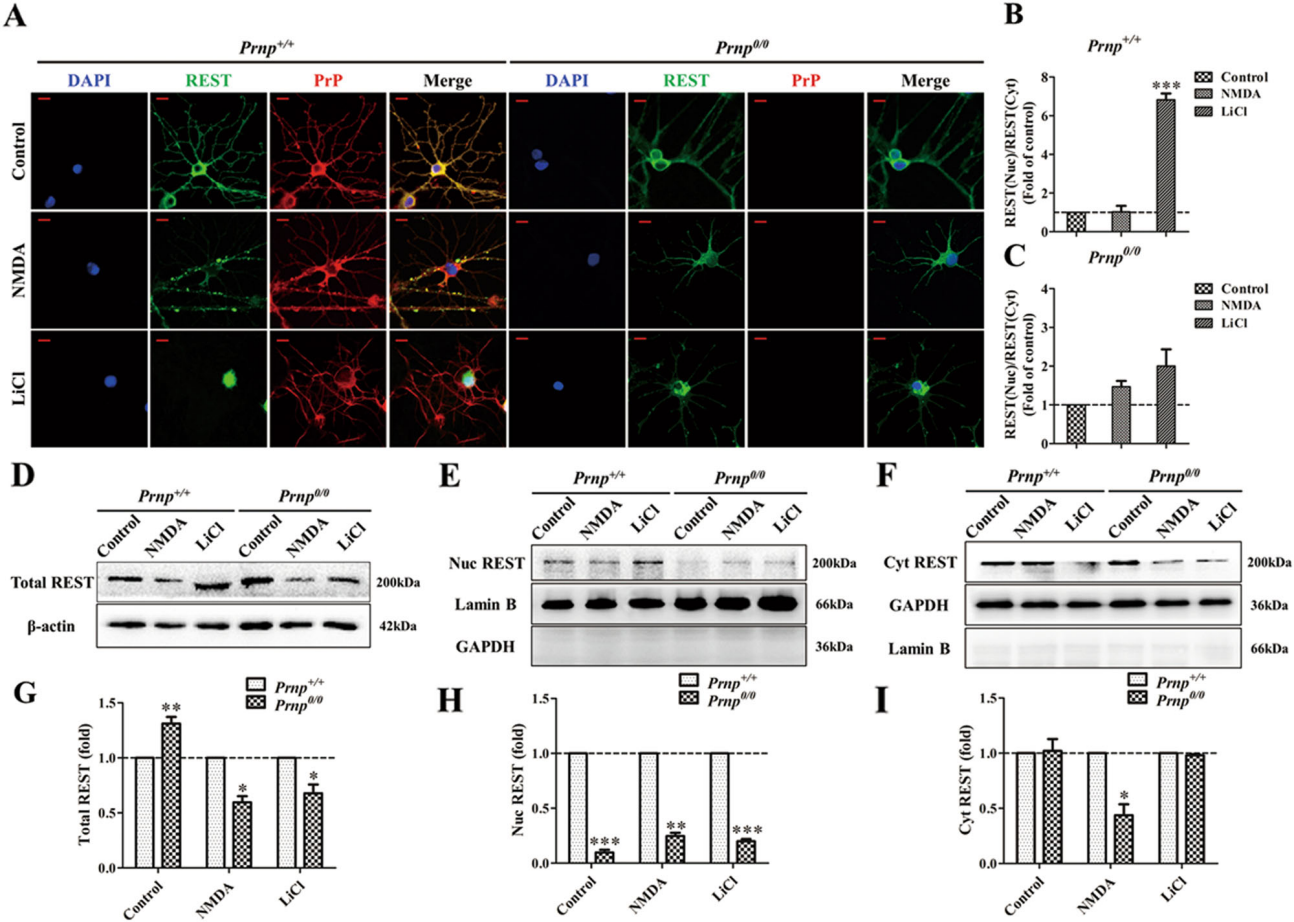
PrP^C^ regulates the functional translocation of REST. **a** Representative double-staining confocal immunofluorescent images of *Prnp*^*+/+*^ and *Prnp*^*0/0*^ mice primary hippocampal neurons for REST (green) and PrP^C^ (red) in each group without treatment or treated with NMDA or LiCl. Nuclei (blue) are stained with DAPI. Scale bars = 10 μm. **b**, **c** Fluorescence quantitative analyses of the ratio of REST in the nucleus to REST in the cytoplasm in *Prnp*^*+/+*^ and *Prnp*^*0/0*^ (**a**). Fluorescence intensity was normalized to each control (dashed lines) and ***P < 0.001 vs the control. **d**-**f** Immunoblotting confirms the total amount, nuclear amount and cytoplasmic amount of REST in *Prnp*^*+/+*^ and *Prnp*^*0/0*^ groups, as indicated. Nuclear and cytoplasmic fractions were collected separately and the fractions immunoblotted for REST. The nucleus-localized proteins Lamin B and GAPDH demonstrate separation of the nuclear and cytoplasmic fractions. **g** Quantitative analyses of (**d**); **h** Quantitative analyses of (**e**); **i** Quantitative analyses of (**f**). Total REST normalized to β-actin. Nuclear and cytoplasmic REST are normalized to Lamin B and GAPDH, respectively, and expressed as a ratio to the corresponding data in *Prnp*^*+/+*^. Data are presented as means ± SD of triplicate experiments. *P < 0.05; **P < 0.01; ***P < 0.001 vs *Prnp*^*+/+*^

Previous reports have shown that activation and translocation of REST is a universal feature in response to stressors^25^. Nuclear REST is a key factor, not only for developmental switch of NMDARs under physiological conditions, but also for neuroprotection in neurodegenerative diseases, such as prion diseases^23,24^ and AD^22^. Thus, to further study the potential effect of PrP^C^ on REST, we compared the expression and distribution of REST in primary cultured WT and *Prnp*^*0/0*^ hippocampal neurons by immunofluorescence (IF) and western blot (WB) analysis. In the WT group, PrP^C^ colocalized with REST in the cytoplasm under normal conditions. *N*-methyl-d-aspartic acid (NMDA), a selective NMDAR agonist (10 μM, 24 h), stimulated the accumulation of REST partly in synapses, and a larger increase of PrP^C^ in the soma. Lithium chloride (LiCl), a selective REST agonist (10 mM, 48 h), remarkably induced the nuclear translocation of REST (Fig. 3a, b) (Fig. S2), a result consistent with our previous report. However, in the absence of PrP^C^, REST had very little response to the agonist and most of it located in the cytoplasm (Fig. 3a, c). Quantitative WB analysis revealed results consistent with IF (Fig. 3d–i). Although total REST in the PrP-null group was 1.31-fold higher than in the WT group under normal conditions, total REST significantly decreased by 40.41% and 32.22% after exposure to NMDA and LiCl, respectively (Fig. 3d, g). What’s more, in the *Prnp*^*0/0*^ groups, compared with the control, total REST decreased significantly more, by 45.44% and 51.62% in the NMDA and LiCl, respectively (Fig. S3). Nuclear REST was markedly lost in each group when PrP^C^ was absent (Fig. 3e, h). This demonstrates that the functional translocation of REST in response to NMDA and LiCl treatment depends on the presence of PrP^C^.

### PrP^C^ is essential for REST-dependent NMDAR expression

**Fig. 4:**
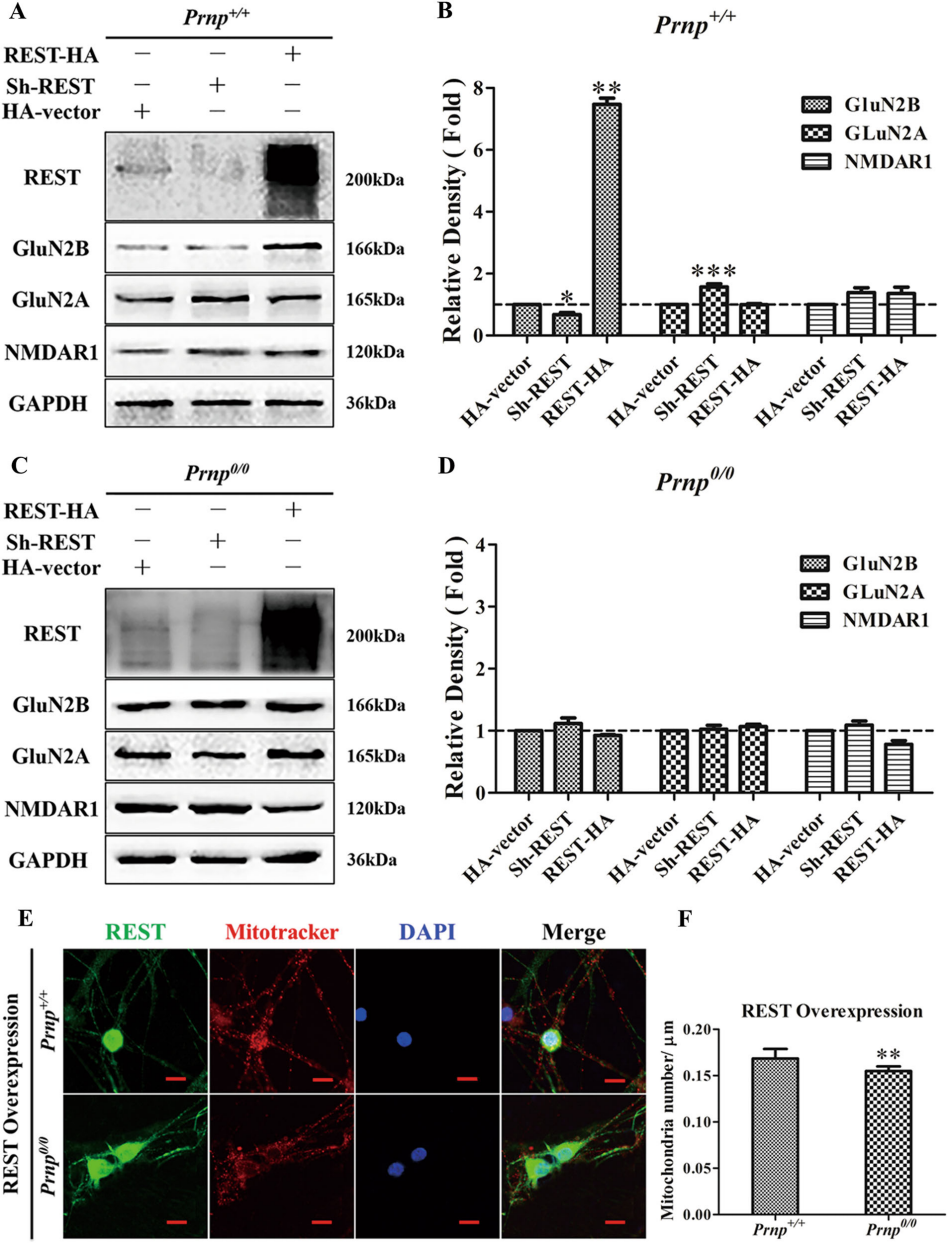
PrP^C^ is essential for REST-regulated expression of NMDARs and mitochondrial numbers. *Prnp*^*+/+*^ **a** and *Prnp*^*0/0*^ **c** mice primary hippocampal neurons were transfected with a control HA vector, or REST shRNA (REST knockdown) or REST-HA vector (REST overexpression). Cellular proteins were immunoblotted for REST, GluN2B, GluN2A and NMDAR1. **b** and **d** quantitative analyses of **a** and **c**, respectively. Immunoblot density in **b** and **d** were normalized to GAPDH and expressed as the relative density to the HA vector (dashed lines) in each group. *P < 0.05; **P < 0.01; ***P < 0.001 in (**b**) vs the HA vector group. **e** Confocal immunofluorescence labeling for total REST (green), including exogenous (overexpression of REST) and endogenous REST, and mitochondria (MitoTracker Red) in *Prnp*^*+/+*^ and *Prnp*^*0/0*^ hippocampal neurons. Nuclei (blue) were stained with DAPI. Scale bars = 10 μm. **f** Quantification of mitochondrial number (**e**) in a segment of neuronal process 200 μm length beginning from the cell body of neurons (*n* = 30). Experiments were performed in triplicate. **P < 0.01; ***P < 0.001 vs *Prnp*^*+/+*^

REST provides a regulatory hub that coordinately regulates multiple physiological and pathological pathways of neuronal development and neurological diseases in vitro and in vivo^10^. Although REST-dependent epigenetic remodeling is critical to ischemia-induced neuronal death^26^, other reports reveal that overexpression of REST plays a critical neuroprotective role^22,24^. Therefore, we examined whether overexpression or knockdown of REST could functionally recover NMDARs expression in the absence of PrP^C^. Thus, we examined the expression of GluN2B, GluN2A and NMDAR1 when REST was blocked or overexpressed in WT or PrP-null neurons. Importantly, overexpression of REST significantly increased the level of GluN2B by 7.47-fold compared with the HA vector control in the WT group. Conversely, knockdown of REST markedly suppressed the level of GluN2B by 32.16% but increased the expression of Glu2A 1.57-fold compared with WT control (Fig. 4a, b). By contrast, neither knockdown nor overexpression of REST had any significant effect on NMDAR expression (Fig. 4a, b). This strongly suggests that PrP^C^ is essential for REST-dependent NMDAR expression, especially for GluN2B. Additionally, the number of normal mitochondria, mediated by the neuroprotective function of REST^24^, decreased (Fig. 4e, f). Even though REST was overexpressed, compared with the WT group, the mitochondrial number decreased by 7.92% (Fig. 4e, f) in the *Prnp*^*0/0*^ group, implying that the presence of PrP^C^ might have an effect on the REST-mediated density of mitochondria.

### REST effects on mitochondria partially depend on PrP^C^

It has been shown that Aβ induces an extrasynaptic NMDAR-dependent increase in nitric oxide, leading to mitochondrial dysfunction and synapse deficiency^27,28^. Overexpression of REST in primary cortical neurons alleviates neurotoxicity peptide (PrP106-126)-induced neuronal oxidative stress and mitochondrial damage^24^. Importantly, loss of PrP^C^ results in decreased mitochondrial numbers and abnormal mitochondrial morphology^29^. In light of these previous studies, we further explored the potential relationship of PrP^C^ with REST-regulated mitochondrial numbers and distribution.

**Fig. 5:**
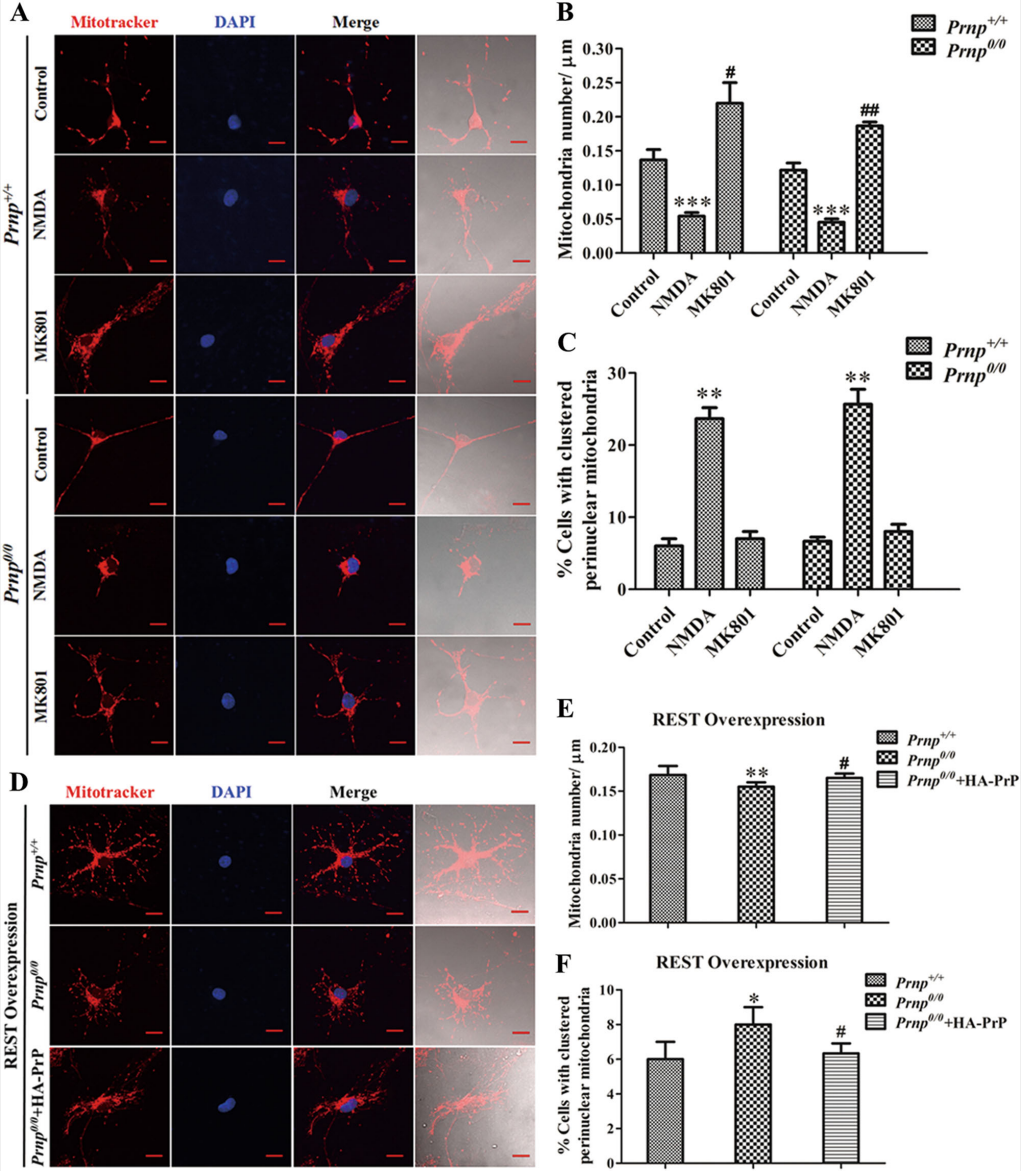
PrP^C^ effects on the density and distribution of mitochondria. **a** Following treatment with NMDA or MK-801 (or no treatment), *Prnp*^*+/+*^ and *Prnp*^*0/0*^ mice primary hippocampal neurons were stained with MitoTracker Red to visualize mitochondria and analyzed by fluorescence microscopy. Nuclei (blue) are stained with DAPI. Scale bars = 10 μm. **b** Quantification of mitochondrial number (**a**) in a segment of neuronal process 200 μm in length beginning from the cell body of neurons (*n* = 30). ^#^*P* < 0.05; ^##^*P* < 0.01; ***P < 0.001 *vs* corresponding control. **c** Quantification analysis of (**a**) to confirm the number of cells harboring perinuclearly clustered mitochondria. For this quantification, mitochondria of at least 500 cells per experiment were determined in a blinded manner. Quantifications were based on triplicates of at least three independent experiments. **P < 0.01 vs corresponding control. **d** Co-transfection of PrP^C^ and REST rescued the density and distribution of mitochondria. Scale bars = 10 μm. **e** Quantification of mitochondrial number (**d**) in a segment of neuronal process 200 μm in length beginning from the cell body of neurons (*n* = 30). **P < 0.01 vs *Prnp*^*+/+*^. ^#^*P* < 0.05 is *Prnp*^*0/0* ^+ HA-PrP *vs Prnp*^*0/0*^. **f** Quantification analysis of (**d**) to confirm the number of cells harboring perinuclearly clustered mitochondria. For this quantification, mitochondria of at least 500 cells per experiment were determined in a blinded manner. Quantifications were based on triplicates of at least three independent experiments. *P < 0.05 vs *Prnp*^*+/+*^. ^#^*P* < 0.05 is *Prnp*^*0/0* ^+ HA-PrP *vs Prnp*^*0/0*^

The NMDAR antagonist dizocilpine maleate (MK-801) improves mitochondrial function and energy status^30^, linking NMDAR activation and mitochondrial function^31^. WT and *Prnp*^*0/0*^ hippocampal neurons were treated with either NMDA (10 μM, 24 h) or MK-801 (Dizocilpine) (10 μM, 24 h) to observe the density and distribution of mitochondria (Fig. 5a–c). In both the WT and *Prnp*^*0/0*^ groups, NMDA significantly decreased mitochondrial numbers by 60.49% and 63.01%, respectively, compared with controls. MK-801 markedly increased mitochondrial numbers in the WT and *Prnp*^*0/0*^ groups by 1.61-fold and 1.53-fold, respectively, relative to corresponding control.

Exposure of hippocampal neurons to NMDA significantly increased the number of cells harboring clustered perinuclear mitochondria in both WT and *Prnp*^*0/0*^ groups, whereas the number in the latter group increased slightly. As overexpression of REST had a diminished effect on mitochondrial numbers in the *Prnp*^*0/0*^ group compared with the WT group, (mitochondrial number decreased by 7.92%), through a PrP^C^ rescue experiment we directly explored the role of PrP^C^ in REST-regulated mitochondria. A REST-HA vector was co-transfected with a HA vector into WT or *Prnp*^*0/0*^ hippocampal neurons, or co-transfected with a PrP-HA vector into *Prnp*^*0/0*^ hippocampal neurons. As expected, co-overexpression of PrP^C^ and REST in *Prnp*^*0/0*^ cultures partially restored mitochondrial numbers compared with the *Prnp*^*0/0*^ group transfected with REST alone (Fig. 5d, e). Moreover, the number of cells harboring clustered perinuclear mitochondria also significantly decreased when PrP^C^ and REST were co-transfected into *Prnp*^*0/0*^ mice (Fig. 5d, f).

### PrP^C^ plays a critical role in REST-regulated GluN2A and GluN2B expression

**Fig. 6:**
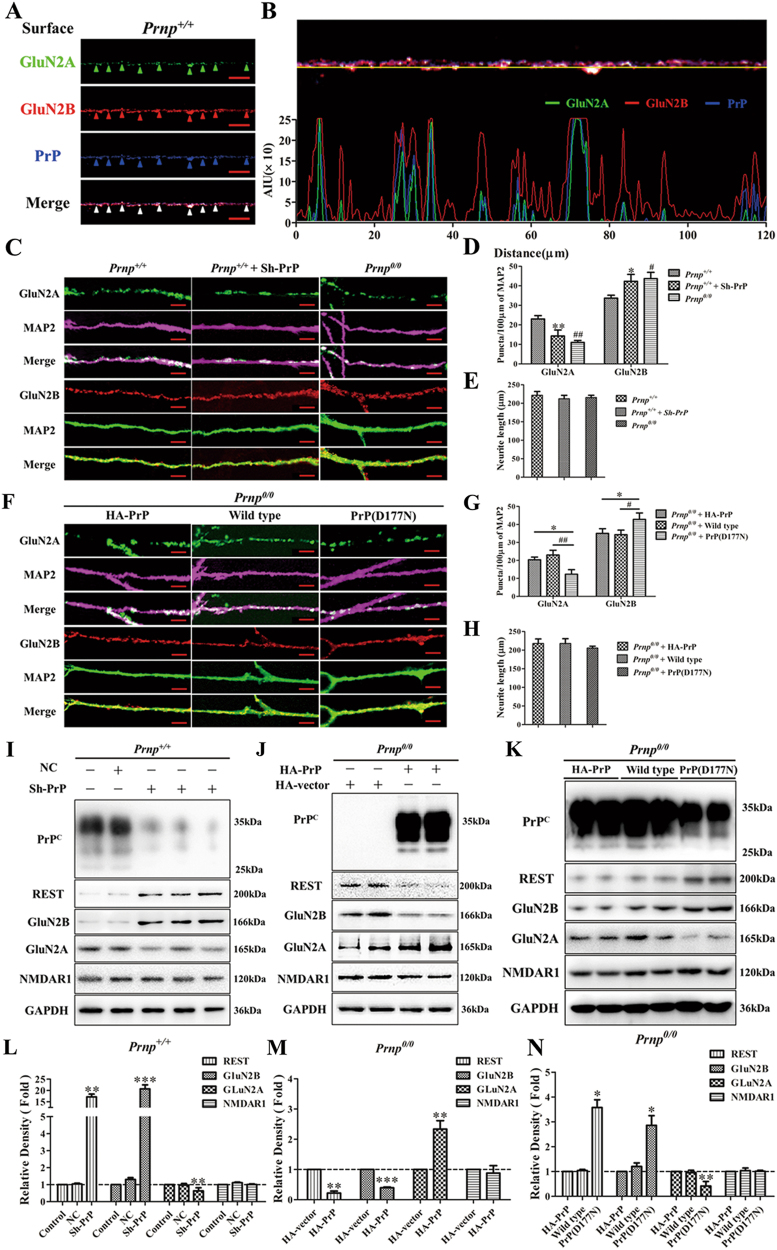
PrP^C^ plays a critical role in REST-regulated expression levels of GluN2A and GluN2B. **a** Triple-labeled WT mouse hippocampal neurons for GluN2A (green), GluN2B (red) and PrP^C^ (blue) along dendritic processes. The arrowheads highlight examples of clear colocalization of GluN2A (green), GluN2B (red), and PrP^C^ (blue). Scale bars = 10 μm. The yellow line in the top panel of (**b**) indicates the position in (**a**) of the line scan shown in the bottom panel of (**b**). **c**-**e** Knockdown of PrP^C^ disturbed the density of GluN2A and GluN2B. **f**–**h** Overexpression of full-length PrP^C^ restores *Prnp*^*0/0*^ induced disorder of GluN2A and GluN2B. **c**, **f** Microtubule-associated protein (MAP2) is the specific dendritic maker. GluN2A (green) protein puncta along processes (MAP2, magenta) and GluN2B (red) protein puncta along processes (MAP2, green) in neurons of *Prnp*^*+/+*^, *Prnp*^*+/+*^ + Sh-PrP and *Prnp*^*0/0*^, or *Prnp*^*0/0*^ culture transfected with HA-PrP vector, wild-type PrP vector or PrP(D177N) vector. Scale bars = 10 μm. **d**, **g** Quantifications of GluN2A and GluN2B puncta density are normalized to 100 μm neurite length, as assessed by MAP2 staining. In (**d**), *P < 0.05; **P < 0.01 is *Prnp*^*+/+*^ + Sh-PrP *vs Prnp*^*+/+*^, ^#^*P* < 0.05; ^##^*P* < 0.01 is *Prnp*^*0/0*^ *vs Prnp*^*+/+*^. In (**g**), *P < 0.05 is *Prnp*^*0/0* *+* ^PrP(D117N) *vs Prnp*^*+/+*^, ^#^*P* < 0.05; ^##^*P* < 0.01 is *Prnp*^*0/0* *+* ^PrP(D117N) *vs Prnp*^*0/0* *+* ^wild-type PrP. **e**, **h** Average neurite length for each randomly tested neuron in each group (*n* = 30). **i** Immunoblot of REST, GluN2A, and GluN2B in *Prnp*^*+/+*^ neurons pre-transfected with negative control vector (NC) or three separate PrP^C^ shRNAs (Sh-PrP; each lane represents a different shRNA). **l** Quantitative analyses of (**i**). **j** Immunoblot of REST, GluN2A, and GluN2B in *Prnp*^*0/0*^ neurons pre-transfected with HA vector or HA-PrP vector. **m** Quantitative analyses of (**j**). **k** Immunoblot of REST, GluN2A and GluN2B in *Prnp*^*0/0*^ neurons pre-transfected with HA-PrP vector, or wild-type PrP vector or PrP(D117N) mutant vector. **n** Quantitative analyses of (**k**). Immunoblot density normalized to GAPDH and expressed as a relative density to the control group in (**i**), or to the HA vector-transfected group in (**j**), or to the HA-PrP vector-transfected group in (**k**). Data are presented as means ± SD in triplicate experiments. In (**l**), **P < 0.01; ***P < 0.001 *vs* control. In (**m**), **P < 0.01; ***P < 0.001 *vs* HA vector-transfected group. In (**n**), *P < 0.01; **P < 0.01 *vs* HA-PrP vector-transfected group

Previous reports have demonstrated that native PrP^C^ mediates an important neuroprotective role through its ability to inhibit NR2D subunits^16^. Although NR2B subunits did not immunoprecipitate with PrP^C^ in that study, PrP^C^ may interact with NMADRs subunits through other and/or indirect ways, an interaction that needs further exploration. So, to examine whether there is an association between NR2 subunits and PrP^C^, we analyzed the surface expression of Glu2A, Glu2B, and in WT hippocampal neurons using immunolabel reactivity (Fig. 6a). Although Glu2A expression was slight, Glu2B and PrP^C^ could well localize in the place where Glu2A existed (Fig. 6b). We then stained total Glu2A and total Glu2B along dendritic processes in WT and *Prnp*^*0/0*^ neurons. The specific dendritic marker, microtubule-associated protein (MAP2) was used to reveal dendrites by IF. Glu2A-positive and Glu2B-positive puncta along processes were quantitatively analyzed. PrP^C^ rescue experiments were also used to explore the function of PrP^C^ by IF and WB. First, WT cultures were transfected with three different short hairpin RNA (shRNA)-mediated PrP knockdown vectors (*Prnp*^*+/+*^ + Sh-PrP) and compared with the PrP-null cultures (*Prnp*^*0/0*^) to exclude the interfering factor of genetic background in the knockout mice. In a second experiment, PrP-null cultures were transfected with HA-PrP vector (*Prnp*^*0/0* ^+ HA-PrP) to observe the function of full-length PrP^C^. Third, PrP-null cultures were transfected with a WT PrP^C^ vector^32^(*Prnp*^*0/0* ^+ WT) and compared with the HA-PrP vector group to remove the potential influence of the HA tag to the location of PrP^C^ and to further confirm the function of full-length PrP^C^. In a final experiment, PrP-null cultures were transfected with a PrP^C^ mutant (D177N point mutation)^32^ [*Prnp*^*0/0* ^+  PrP(D177N)] that is less efficiently trafficked to the surface than the WT PrP and accumulates in the cytoplasm even without proteasome inhibition. These cultures were compared with the WT PrP^C^ to further confirm the role of full-length PrP^C^. Remarkably, both in the *Prnp*^*+/+*^ + Sh-PrP group and in the *Prnp*^*0/0*^ group, Glu2A puncta significantly decreased by 37.70% and 52.17% relative to the WT group (23.07 ± 0.05 per 100 μm of dendrites), respectively. Conversely, Glu2B was present in more continuously abundant puncta, increased 1.25-fold (*Prnp*^*+/+*^ + Sh-PrP) and 1.30-fold (*Prnp*^*0/0*^) over the WT group (33.67 ± 0.06 per 100 μm of dendrites) (Fig. 6c, d). On the other hand, overexpression of HA-PrP vector or WT PrP vector had similar effects. They both partly restored the densities of GluN2A puncta (*Prnp*^*0/0*^ versus *Prnp*^*0/0*^ + HA-PrP: 11.01 ± 0.07 versus 20.33 ± 0.09 per 100 μm of dendrites; *Prnp*^*0/0*^ versus *Prnp*^*0/0* ^+  WT PrP: 11.01 ± 0.07 versus 23.03 ± 0.05 per 100 μm of dendrites) and reduced the densities of GluN2B puncta (*Prnp*^*0/0*^ versus *Prnp*^*0/0*^ + HA-PrP: 43.67 ± 0.04 versus 35.07 ± 0.07 per 100 μm of dendrites; *Prnp*^*0/0*^ versus *Prnp*^*0/0* ^+ WT PrP: 43.67 ± 0.04 versus 34.33 ± 0.05 per 100 μm of dendrites). However, the D177N mutant had no significant effect on the expression of GluN2A (12.33 ± 0.07 per 100 μm of dendrites) or GluN2B (42.83 ± 0.03 per 100 μm of dendrites) (Fig. 6f, g). Total neurite length (the cumulative length of all neurites of a single neuron) was not significantly different in the groups tested (*n* *=* *30* randomly selected neurons per coverslip within each experiment. Each experiment was repeated with, at least, three independent cultures). The average length of neurites in each group is shown in Figure. 6e, h.

Consistently, quantitative WB analysis revealed the following. (1) Knockdown of PrP^C^ in WT culture remarkably induced the expression of REST (increased to 17.14-fold of the *Prnp*^*0/0*^ group), stimulated the level of GluN2B (increased to 20.68-fold of the *Prnp*^*0/0*^ group) and suppressed the level of GluN2A (decreased to 63.54% of the *Prnp*^*0/0*^ group). However, NMDAR1 expression was not significantly different, and negative control vector (NC) showed similar results to control group (Fig. 6i, l). (2) PrP^C^ overexpression significantly inhibited the expression of REST (decreased to 21.94% of the *Prnp*^*0/0*^ group), contributing to the suppression of GluN2B (decreased to 39.50% of the *Prnp*^*0/0*^ group) and the promotion of GluN2A (increased to 2.33-fold of the PrP-null group). NMDAR1 expression was not significantly different in the two groups (Fig. 6j, m). (3) Both HA-PrP vector and WT PrP vector had similar degrees of functional action to REST, GluN2B and GluN2A. However, PrP (D177N) mutant failed to recover the expression of these proteins in *Prnp*^*0/0*^ culture (Fig. 6k, n). Overall, our data indicate a critical and novel relationship between full-length PrP^C^, GluN2A and GluN2B. This is mediated by REST to maintain the correct proportions of NMDAR subunits.

## Discussion

### The importance of a developmental switch maintaining the correct ratio of GluN2B-to-GluN2A

NMDARs are necessary regulators of brain plasticity^1^. During the development of synapse structure and function, they transform precise patterns of neuronal activity into long-term changes that are thought to underlie higher cognitive functions^33^. Moreover, NMDARs switch their subunits from predominantly GluN2B to primarily GluN2A during early postnatal development^34^. This subunit switch is evolutionarily conserved from amphibians to mammals and occurs all over the CNS during a time window coinciding with synapse growth and neuronal circuitry refinement^1,33^. However, the mechanisms responsible for the GluN2B-to-GluN2A subunit exchange have yet to be fully defined^12^. In neurodegenerative diseases, recent studies also indicate that GluN2B-containing NMDARs have an essential role in mediating the adverse effects of Aβ^27^. GluN2B antagonists can rescue Aβ-induced damage of long-term potentiation and synaptic impairment^35^. However, all of the clinical trials of first-generation NMDAR antagonists were disappointing as a result of unendurable side-effects and short therapeutic windows^36^. Another potential limitation is the lack of subunit selectivity of the drugs^2,35^. GluN2B-specific antagonists offer significant neuroprotection with a better side-effect profile. Conversely, activation of GluN2A may exhibit pro-survival effects^37^ via CREB signaling, although the neuroprotective role of GluN2A is still controversial^38,39^. Additionally, in patients with Parkinson’s disease, the degeneration of nigral dopaminergic neurons gives rise to overactivation of glutamatergic projections^40,41^. In the striatal membrane of l-3,4-dihydroxyphenylalanine (l-DOPA)-treated dyskinesia animals, the increased synaptic abundance of GluN2A and redistribution of GluN2B from synaptic to extrasynaptic regions demonstrate that selectively targeting specific NMDARs might be more hopeful^42^. Understanding the precise molecular mechanisms responsible for the GluN2B-to-GluN2A exchange could provide new perspectives for the development of therapeutic strategies.

### PrP^C^ plays a novel and critical role in the REST-dependent developmental switch in synaptic NMDA receptors

During embryogenesis in pluripotent stem cells and neural progenitors, REST is a widely expressed gene-silencing factor. REST plays an important role for synaptic function via epigenetic remodeling by silencing coding and noncoding neuronal genes^10^. At the gene level, REST takes part in the postnatal switch in synaptic NMDARs by reducing GluN2B expression through epigenetic remodeling of *Grin2b*^43^. Some questions remain unanswered, such as what mechanism turns on REST expression in differentiated neurons, and which factor regulates the long-term increase in GluN2A expression during postnatal development.

Mature PrP^C^ is a glycoprotein attached by a carboxyl(C)-terminal glycosylphosphatidylinositol (GPI) anchor to the extracellular leaflet of the plasma membrane^44^. PrP^C^ can undergo different types of physiological cleavage, producing N2 and C2 fragments^13^. PrP^C^ contacts with, and signals through, multiple cell surface proteins and signaling pathways. This highlights the need for a better understanding of the mechanisms of PrP^C^ interaction with its binding factors, both physiologically and pathologically^44^. Comparing WT and *Prnp*^*0/0*^ revealed that PrP^C^ expression at synapses contributes to hippocampal synaptic function and exhibits neuroprotection by regulating neuronal excitability. In particular, PrP^C^ requires copper to facilitate S-nitrosylation-mediated NMDAR suppression^17^. However, GluN2B subunits did not immunoprecipitate with PrP^C^ in a previous study^16^ and no studies have yet explored the relationship between PrP^C^ and GluN2A. PrP^C^ might interact with GluN2B through indirect ways, an aspect needing further research. Nucleocytoplasmic transport is thought to be important for REST as a transcriptional repressor regulating neuronal gene expression^22,25^. In this study, nuclear REST was markedly lost in the absence of PrP^C^, demonstrating that the functional translocation of REST in response to NMDA and LiCl treatment depends on the presence of PrP^C^. Overexpression of exogenous PrP^C^ rescued excessive REST induced GluN2A and GluN2B imbalance by increasing the expression of GluN2A and inhibiting the level of GluN2B in PrP-null neurons. To our knowledge, our study is the first to demonstrate that PrP^C^-mediated REST-dependent expression of GluN2A and GluN2B contributes to maintain the correct subunit proportions of NMDARs and cellular homeostasis, deserving to be further explored as a novel and viable therapeutic target against dysfunctions of glutamatergic transmission.

## Materials and methods

### Animals

*PrnP*^*0/0*^ mice on a C57BL/6J × 129Sv genetic background were kindly supplied by Dr. Charles Weissmann^45,46^. Control C57BL/6J mice with no genomic modifications (WT), 6–8 weeks of age and weighing 18–25 g, were obtained from Beijing Experimental Animal Center. Mice were housed 3–4 per cage and under controlled environmental conditions (21–23 °C; 40–60 % humidity; 12-h light/dark cycle) with free access to water and standard pelleted food. All animal experiments were conducted in accordance with the guidelines of Beijing Municipality on the Review of Welfare and Ethics of Laboratory Animals and approved by the Beijing Municipality Administration Office of Laboratory Animals (BAOLA).

### Plasmids and transfection

The pCMV-HA-Rest vector of full-length REST (cat no. PPL50007-2a)^24,25^ was obtained from Public Protein/Plasmid Library (Nanjing, China, GeneShare Technology, Co, Ltd). The pGPH1/GFP/Neo-REST-Mus shRNA vectors^24,25^, the pGPH1/GFP/Neo-PRNP-Mus shRNA vectors (Table 1) and the negative control vector (NC)—which has the same number of corresponding bases as shRNA-REST or shRNA-PRNP but does not target any known gene—were all obtained from GenePharma (Suzhou, China). The full-length mouse prion protein (PRNP) complementary DNA (cDNA) was originally cloned from total brain of C57BL/6J mice by PCR. The primers were designed according to the gene sequence of Mus musculus prion protein (PRNP) mRNA in GenBank (Gene ID: 19122). Oligonucleotides 5′-CGGAATTCATGGCGAACCTTGGCTACTG-3′ (forward) and 5’-GCCTCGAGTCACATGTGCTTCATGTTGGTTTTTCCCACGATCAGGAAGATGA-3′ (reverse) were used to introduce *Eco*RI and *Xho*I sites flanking the cDNA (The underlined alphabets denote the "Restriction enzyme cutting sites" of EcoRI and XhoI, respectively.). The PCR product was cloned into vector pCMV-HA (Clontech, Kyoto, Japan) to generate plasmid pCMV-HA-PRNP (HA-PrP) by standard molecular biology techniques and confirmed by sequencing. All the primers were synthesized by Sangon Company (Shanghai, China). Full-length mouse PrP with 3F4 epitope (WT PrP^C^) (Addgene plasmid # 13917) and moPrP(3F4) D177N (Addgene plasmid # 1319) were gifts from Susan Lindquist^32^. For transfection^23,24,47,48^, cultured primary hippocampal neurons were washed with Opti-MEM (Gibco) and then transfected with the appropriate plasmids using the Lipofectamine^TM^ Ltx & Plus^TM^ Reagent (Thermo Fisher, Waltham, MA, USA) in Opti-MEM according to the manufacturer’s instructions. The amounts of plasmids and reagents were 2 μg and 3 μl per 12-well, respectively. Forty-eight hours after transfection, cells were observed using an upright fluorescence confocal microscopy (Olympus, Tokyo, Japan) or subjected to immunoblot analyses.

**Table 1:** Sequences of REST-targeting shRNA(Sh-REST), PRNP-targeting shRNA(Sh-PrP)

Name	Sequence (5′→3′)
*REST*-mediated shRNA:	
Target sequence 1	GCTGTGGCTACAATACCAACC
Target sequence 2	GTGCAATTATGTGGCCTCTAA
Target sequence 3	GGATTCACAGCGCTAAGAAGT
*PRNP*-mediated shRNA:	
Target sequence 1	GCAACCGTTACCCACCTCAGG
Target sequence 2	GCCTATTACGACGGGAGAAGA
Target sequence 3	GTGACTATGTGGACTGATG

### Statistical analysis

All assays were performed at least three times. Data are expressed as means ± S.D. For in vivo experiments, six hippocampal homogenates were separately collected and tested in each group. One-way ANOVA, followed by *Dunnett’s* test was performed for whole hippocampal lysate experiments in Figure 1^11^. Two-way ANOVA was performed for Figure 2. Other comparisons for parametric data were made using Student’s test or one-way ANOVA followed by *post hoc* Turkey’s test or two-way ANOVA test using the SPSS software (version 13.0: SPSS Inc., Chicago, IL, USA), GraphPad Prism 5 software (La Jolla, CA, USA) and Image J (National Institutes of Health, USA). *P* *<* *0.05* was considered statistically significant^24,25^.

## Electronic supplementary material

Fig. S1

Fig. S2

Fig. S3

Supplementary Information
