# Supplementary figures and images for: Prion protein is essential for the RE1 silencing transcription factor (REST)-dependent developmental switch in synaptic NMDA receptors
Source: Cell Death Dis. 2018 May 10;9(5):541. doi: 10.1038/s41419-018-0576-z (PMC5945644; doi:10.1038/s41419-018-0576-z)

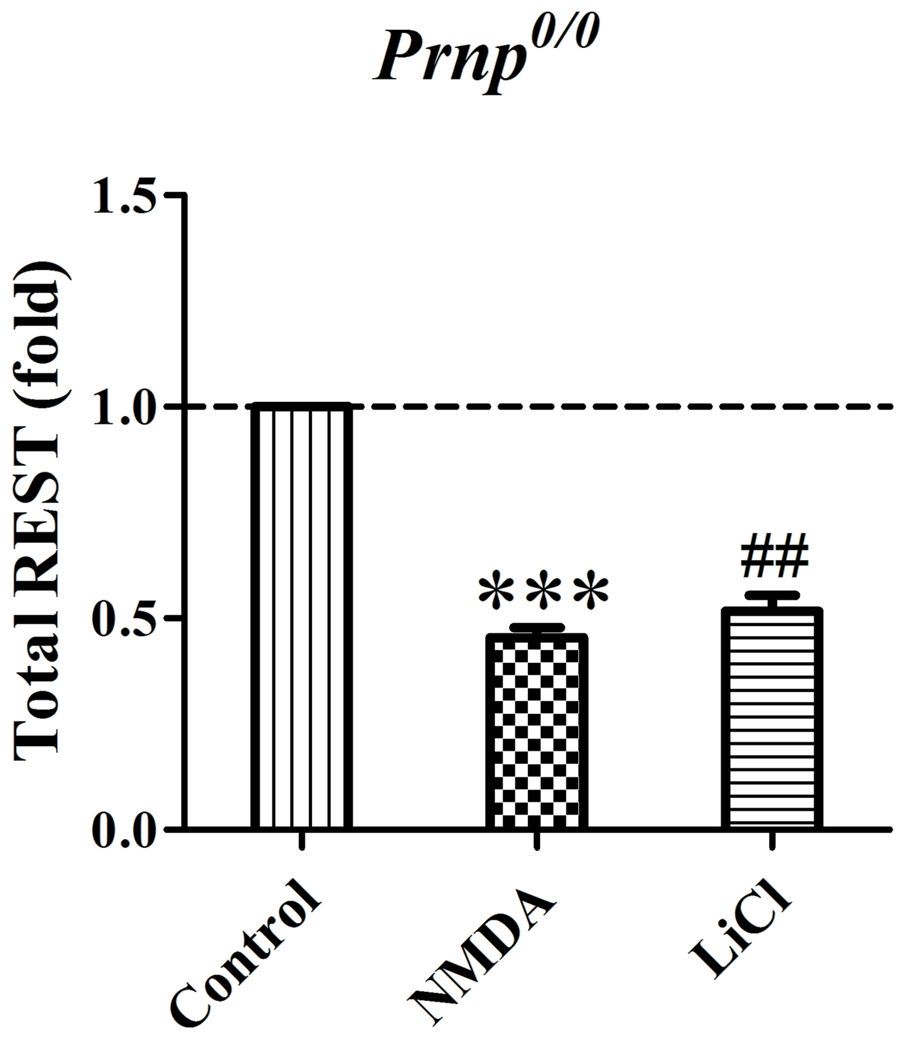

Supplement: Supplementary file 3 — Fig. S3 [file 41419_2018_576_MOESM3_ESM.tif]
