# Supplementary material for: Prion protein is essential for the RE1 silencing transcription factor (REST)-dependent developmental switch in synaptic NMDA receptors
Source: Cell Death Dis. 2018 May 10;9(5):541. doi: 10.1038/s41419-018-0576-z (PMC5945644; doi:10.1038/s41419-018-0576-z)
Supplement: Supplementary file 4 — Supplementary Information [file 41419_2018_576_MOESM4_ESM.docx]

**Supplementary Materials for**

**Prion protein is essential for the RE1 silencing transcription factor (REST)-dependent developmental switch in synaptic NMDA receptors**

**Reagents**

The rabbit polyclonal anti-REST antibody (07-579) (1:500) was purchased from Millipore (MA, USA). The goat polyclonal anti-NRSF antibody (P-18) (sc-15118) (1:50 for IF), mouse monoclonal anti-PrP antibody (AH6) (sc-69896) (1:200) were purchased from Santa Cruz Biotechnology (Santa Cruz, CA, USA).The rabbit polyclonal anti-REST antibody (22242-1-AP) (1:200), rabbit polyclonal anti-NMDAR2A antibody (19953-1-AP) (1:200; WB), (1:50; IF), rabbit polyclonal anti-NMDAR2B antibody (21920-1-AP) (1:200; WB), (1:50; IF), rabbit polyclonal anti-beta-Catenin antibody (51067-2-AP) (1:200), rabbit polyclonal anti-GSK3β antibody (22104-1-AP) (1:200)，mouse monoclonal anti-GAPDH antibody (60004-1-lg) (1:1000), rabbit polyclonal anti-Lamin B1 antibody (12987-1-AP) (1:500) were purchased from Proteintech Biotechnology (Chicago, IL, USA).The rabbit monoclonal anti-NMDAR1 antibody (D65B7) (1:500), rabbit polyclonal anti-p-β-Catenin antibody (Ser33/37) (1:500), rabbit monoclonal anti-p-GSK-3β antibody (Ser9) (D85E12) (1:500) were purchased from cell signaling technology (Danvers, MA, USA). The mouse monoclonal anti-MAP2 antibody (AP20) (MA5-12823) (1:200) and donkey anti-mouse IgG (H+L) highly cross-adsorbed secondary antibody (Alexa Fluor 350) (1:500) (A10035) were purchased from Thermo Fisher (Waltham, MA, USA). Anti-NMDA Receptor 2A (GluN2A) (extracellular)-ATTO-488 (AGC-002-AG) (1:50), anti-NMDA Receptor 2B (GluN2B) (extracellular)-ATTO-594 (AGC-003-AR) (1:50) were purchased from Alomone labs (Jerusalem, Israel). The rabbit polyclonal anti-rat β-actin antibody (AP0060) (1:1000), the goat anti-rabbit IgG (H&L)-HRP secondary antibody (BS13278) (1:5000) were purchased from Bioworld Technology (Nanjing, China Bioworld Technology, Inc.).The Alexa Fluor 488-Conjugated AffiniPure goat anti-rabbit IgG(H+L) (ZF-0511) (1:100), Alexa Fluor 488-Conjugated AffiniPure goat anti-mouse IgG(H+L) (ZF-0512) (1:100), Alexa Fluor 594-Conjugated AffiniPure goat anti-rabbit IgG(H+L) (ZF-0516) (1:100), Alexa Fluor 594-Conjugated AffiniPure goat anti-mouse IgG(H+L) (ZF-0513) (1:100), Peroxidase-Conjugated Affinipure goat anti-rabbit IgG(H+L) (ZB-2301) (1:5000), goat anti-mouse IgG(H+L)(ZB-2305) and rabbit anti-goat IgG(H+L) (ZB-2306) (1:5000) were purchased from Beijing ZSGB Biotechnology (Beijing, China). Rabbit anti-goat IgM/Alexa Fluor 488 antibody (bs-0370R-AF488) (1:100) and rabbit anti-mouse IgG/Alexa Fluor 594 antibody (bs-0296R-AF594) (1:100) secondary antibodies were purchased from Beijing Biosynthesis Biotechnology (Beijing, China). DAPI dihydrochloride was purchased from Beyotime Biotechnology (Shanghai, China). N-Methyl-D-aspartic acid (NMDA) (S7072) and MK-801 (Dizocilpine) (S2857) were purchased from Selleck Chemicals (Houston, TX, USA). Lithium chloride (LiCl) (L4408) was purchased from Sigma-Aldrich (St.Louis, MO, USA). Reagents and apparatus used in immunoblotting assays were purchased from Bio-Rad (Richmond, CA, USA).

**Primary cultured hippocampal neurons**

Dissociated hippocampal neuronal cultures were prepared from postnatal 1-day-old C57BL/6J mice or PrnP0/0 mice, according to the previously described procedure ^1-4^. Briefly, cells were gently dissociated after digestion with papain (Invitrogen, Waltham, MA, USA). The dissociated cells were plated at a final density of 5×105cells/cm2 on polyethyleneimine (Sigma, Milwaukee,WI, USA)-coated plates and cultured in DMEM F12 (Hyclone, Logan, UT, USA), supplemented with 2% B27 (Gibco, Carlsbad, CA, USA), and 0.5% Penicillin-Streptomycin (Gibco). Two days later, 10μM [cytarabine](app:ds:cytarabine) (Sigma) were added to repress the growth of glial cells.

**Protein extraction of hippocampal neurons**

For the extraction of total cell proteins, primary neurons were washed in PBS after treatment or overexpression, and then dissolved and homogenized with pre-chilled RIPA lysis buffer (Beyotime) supplemented with a proteinase inhibitor cocktail (Novasygen, Beijing, China). Homogenates were centrifuged at 12,000 rpm for 10 min at 4 °C, and supernatant was harvested. The supernatant was frozen at −80 °C for western blot analysis to assess the protein levels.

Cytoplasmic and nuclei proteins were extracted using a cytoplasmic and nuclear protein extraction kit (Wuhan Boster Biotech). Protein levels were assessed by western blotting. The blot was stripped and probed with anti-GAPDH (for cytoplasmic extracts) or anti-Lamin B (for nuclear extracts) antibodies.

**Western blotting**

Extracted proteins were separated by SDS-PAGE on 10-15 % gels, and the separated proteins were transferred onto a nitrocellulose membrane. Nonspecific binding sites were blocked by 5 % fat-free dried milk in Tris-buffered saline (TBS-T: 10 mmol/l Tris, 0.15 mol/l NaCL, 0.05 % Tween-20, pH of the solution adjusted to 7.5). Primary antibodies were added and incubated at 4°C overnight. Membranes were washed with TBS-T, and then incubated with the secondary antibody, either goat anti-mouse IgG or anti-rabbit IgG horseradish peroxidase-conjugated antibody (1:5000). Bands of immunoreactive protein were visualized on an image system (Versadoc; Bio-Rad) after membrane incubation with enhanced chemifluorescence (ECF) reagent for 5 minutes.

**Immunofluorescence**

For surface staining of GluN2A, GluN2B and PrPC, coverslips containing cultured neurons were not permeabilized allowing for the selective staining of cell surface protein ^5, 6^. Other processes are according to previously described procedure. Briefly, primary neurons grown on cover slips were washed twice with PBS, fixed by with 4% PFA (P0098) for 15 min, blocked 1h at room temperature by Immunol Staining Blocking Buffer (P0102) and then incubated overnight at 4 °C with the appropriate primary and then secondary antibodies. The nuclei were stained with DAPI.

Immunofluorescence labeling and quantitative analyses for GluN2A and GluN2B immunopositive puncta along MAP2-labeled dendrites were counted as before ^7^. Images of 30 randomly selected neurons per coverslip per condition within each experiment were acquired and each experiment was repeated in at least three independent cultures. Images were analyzed for puncta and neurite length by the ImageJ software.

**Mitochondria density and distribution**

To analyze mitochondrial distribution, treated- hippocampal neurons were incubated with pre-warmed (37℃) staining solution containing the MitoTracker™ Red CMXRos - Special Packaging (M7512) (Invitrogen) (ﬁnal concentration 100 nM) for 45 min ^4^. After mitochondrial staining is complete, the staining solution was replaced with prewarmed fresh media or followed by REST staining as description in the section of Immunofluorescence, and then observed using an upright ﬂuorescence confocal microscope (Olympus, Tokyo, Japan). The number of cells with non-tubular/fragmented mitochondria was determined. All quantiﬁcations were based on at least three independent experiments.

**Preparations of hippocampal homogenates**

The hippocampi were rapidly dissected and snap-frozen in liquid nitrogen. Each group has 6 mice in different time point during hippocampal postnatal development. 6 hippocampal homogenates from 6 mice were separately collected, labelled and examined by WB. Proteins were extracted in RIPA buﬀer containing a cocktail of protease inhibitors (Roche, Basel, Switzerland) and were sonicated for 15 s, and then centrifuged at 20,000 g for 5 min. The supernatants were collected and boiled for 10 min after addition of a loading buﬀer (250 mM Tris-HCl pH 6.8, 10% SDS, 0.5% BPB, 50% glycerol, 0.5 M DTT). The protein concentration of the supernatants was measured using the BCA assay (CWBio). Protein extracts (10 μl) were subjected to SDS-PAGE, and western blotting ^4, 8^.

**References:**

1. Song Z. et al. REST alleviates neurotoxic prion peptide-induced synaptic abnormalities, neurofibrillary degeneration and neuronal death partially via LRP6-mediated Wnt-beta-catenin signaling. *Oncotarget* **7**, 12035-52 (2016).

2. Zhu T. et al. HDAC6 alleviates prion peptide-mediated neuronal death via modulating PI3K-Akt-mTOR pathway. *Neurobiol. Aging* (2015).

3. Song Z. et al. Overexpression of BAT3 alleviates prion protein fragment PrP106-126-induced neuronal apoptosis. *CNS. Neurosci. Ther.* **20**, 737-47 (2014).

4. Song Z. et al. Downregulation of the Repressor Element 1-Silencing Transcription Factor (REST) Is Associated with Akt-mTOR and Wnt-β-Catenin Signaling in Prion Diseases Models. *Front. Mol. Neurosci.* 10, 128 (2017).

5. Tang Y. et al. EGFR signaling upregulates surface expression of the GluN2B-containing NMDA receptor and contributes to long-term potentiation in the hippocampus. *Neurosci.* **304**, 109-21 (2015).

6. Khosravani H. et al. Prion protein attenuates excitotoxicity by inhibiting NMDA receptors. *J. Cell Biology* **181**, 551-65 (2008).

7. Song Z., Yang W., Zhou X., Yang L., Zhao D. Lithium alleviates neurotoxic prion peptide-induced synaptic damage and neuronal death partially by the upregulation of nuclear target REST and the restoration of Wnt signaling. *Neuropharmacology* (2017).

8. Shah S. Z. A. et al. Early Minocycline and Late FK506 Treatment Improves Survival and Alleviates Neuroinflammation, Neurodegeneration, and Behavioral Deficits in Prion-Infected Hamsters. *Neurotherapeutics* 1-21 (2017).

Figure. S1 (A and B) Quantitative analyses of (Fig 1A). (C and D) Quantitative

analyses of (Fig 1C). Immunoblot density in (E–H and I–L) showing quantification of β-catenin (Ser33)/total β-catenin protein, GSK3β (Ser9)/total GSK3β protein, total β-catenin and GSK3β protein normalized to GAPDH. All values were normalized (dashed lines) relative to corresponding data at P3 in each group. Data are presented as means ± SD (n=6). **P < 0.05*; ***P < 0.01*; ****P < 0.001* *vs* corresponding data at P3.

Figure. S2 Lower magnification of representative confocal immunofluorescent images of double-staining of *Prnp^+/+^* mice primary hippocampal neurons for REST (green) and PrP^C^ (red) in each group without treatment or treated with NMDA or LiCl to observe the relationship of PrP^C^ and REST. Scale bars = 20 μm.Figure. S3 Quantitative analyses of total REST (Fig. 3D) in *Prnp^0/0^* groups. Total REST normalized to β-actin and expressed as a ratio to the *Prnp^0/0^* control. Data are presented as means ± SD of triplicate experiments. ****P < 0.001;* ***^##^****P < 0.01* *vs* *Prnp^0/0^*.

Figure. S3 Quantitative analyses of total REST (Fig. 3D) in *Prnp^0/0^* groups. Total REST normalized to β-actin and expressed as a ratio to the *Prnp^0/0^* control. Data are presented as means ± SD of triplicate experiments. ****P < 0.001;* ***^##^****P < 0.01* *vs* *Prnp^0/0^*.
